# Targeting colorectal cancer stem cells using curcumin and curcumin analogues: insights into the mechanism of the therapeutic efficacy

**DOI:** 10.1186/s12935-015-0241-x

**Published:** 2015-10-09

**Authors:** Thamil Selvee Ramasamy, Ain Zubaidah Ayob, Hsu Hsu Lynn Myint, Sharmanee Thiagarajah, Farahnaz Amini

**Affiliations:** Department of Molecular Medicine, Faculty of Medicine, University of Malaya, 50603 Kuala Lumpur, Malaysia; Cell and Molecular Biology Laboratory, Faculty of Medicine, University of Malaya, 50603 Kuala Lumpur, Malaysia; Faculty of Medicine and Health Science, School of Healthy Aging, Medical Aesthetics and Regenerative Medicine, UCSI University, Kuala Lumpur, Malaysia

**Keywords:** Epithelial-mesenchymal transition, MicroRNA, Cancer associated self-renewal signaling pathways, Anti-cancer drug, Chemosensitiser, Drug-/chemo-resistance

## Abstract

Colorectal cancer is one of the commonest cancers in the world and it is also a common cause of cancer-related death worldwide. Despite advanced treatment strategies, the disease is rarely cured completely due to recurrence. Evidence shows that this is due to a small population of cells, called cancer stem cells (CSCs), in the tumour mass that have the self-renewal and differentiation potential to give rise to a new tumour population. Many pre-clinical and clinical studies have used curcumin and its analogues as anti-cancer agents in various types of cancer, including colorectal cancer. Intriguingly, curcumin and its analogues have also recently been shown to be effective in lowering tumour recurrence by targeting the CSC population, hence inhibiting tumour growth. In this review, we highlight the efficacy of curcumin and its analogues in targeting colorectal CSC and also the underlying molecular mechanism involved. Curcumin, in the presence or absence of other anti-cancer agents, has been shown to reduce the size of tumour mass and growth in both in vivo and in vitro studies by affecting many intracellular events that are associated with cancer progression and CSC formation. An insight into the molecular mechanism has unraveled the mode of action via which curcumin could affect the key regulators in CSC, importantly; (1) the signaling pathways, including Wnt/β-catenin, Sonic Hedgehog, Notch and PI3K/Akt/mTOR, (2) microRNA and (3) the epithelial-mesenchymal transition at multiple levels. Therefore, curcumin could play a role as chemosensitiser whereby the colorectal CSCs are now sensitised towards the anti-cancer therapy, therefore, combination therapy using anti-cancer agent with curcumin could be much more effective than treatment using a single cancer agent. This potential treatment modality can be further developed by employing an effective delivery system using a nanotechnology based approach to treat colorectal cancer.

## Overview of colorectal cancer stem cells and curcumin

Colorectal cancer is the third most common cancer, affecting men and women equally [1], and it is the second commonest cause of cancer-related death in the United States and many other developed countries [2, 3]. For patients with advanced colon cancer, the 5-year survival rate is as low as 8 %. It is interesting that, despite the use of aggressive surgical tumour resection and chemotherapy, nearly 50 % of patients with colorectal carcinoma experience a recurrence [4]. Increasing evidence suggests that colorectal cancer cells are driven by a subset of self-renewing cells, termed cancer stem cells (CSCs) or tumour initiating cells, which are distinct from the bulk of the tumour cells [5, 6].

CSC can be defined as cells in a tumour with tumour initiating potential. They can be derived from either self-renewing normal stem cells or from progenitor cells that have acquired the ability of self-renewal due to mutation or the dedifferentiation of mature neoplastic cells [7]. Although they are small in number in a cancer cell mass, with self-renewal and differentiating properties like normal stem cells, they are resistant to eradication by chemotherapy. Hence, targeting these cell populations becomes crucial in the treatment of cancer.

Curcumin, a bioactive compound in the famous Indian spice turmeric, obtained from the plant *Curcuma longa*, is well known for its anti-inflammatory, antioxidant, and antimicrobial activities [8–10]. Besides, curcumin has been shown to have anti-cancer properties [9–12], and its inhibitory effects on various mechanisms have been demonstrated in various human cancer cell lines. Curcumin induces cell death through a variety of mechanisms by targeting pathways, transcription factors, membrane receptors, kinases and cytokines [13].

As currently available conventional treatment modalities for colorectal cancer, which include surgery, radiation, and chemotherapy, are still not effective in curing the disease due to the incidence of secondary cancer or tumour, curcumin and its analogues could provide an effective treatment either as a stand alone or in combination with other chemotherapy drugs. However, there is a need to evaluate the effectiveness of the therapeutic action and its underlying mechanism in order to develop an effective treatment to cure cancer.

### Colorectal cancer

The incidence and rate of mortality associated with colorectal cancer increases with age. The incidence rate of colorectal cancer is known to be 15 times higher in adults aged 50 years and above than in those who are between 20 and 49 years of age [14]. Colorectal cancer incidence and mortality rates are highest in Afro-American populations; incidence rates and mortality rates are, respectively, about 20 and 45 % higher than those in white populations [15]. There is a strong correlation between genetic predisposition and colorectal cancer occurrence. People with a first-degree relative who has had colorectal cancer have two to three times the risk of developing the disease compared to individuals with no family history. If the relative was diagnosed at a young age or if there is more than one affected relative, the risk increases to three to six times that of the general population [16, 17]. About 20 % of all colorectal cancer patients have a close relative who has been diagnosed with the disease [18].

About 5 % of patients with colorectal cancer have a well-defined genetic syndrome that causes the disease [19]. Two genetic syndromes are strongly linked to colorectal cancer; the most common is hereditary non-polyposis colon cancer (HNPCC), also known as lynch syndrome, and familial adenomatous polyposis (FAP) syndrome. HNPCC accounts for 2–4 % of all colorectal cancers, and FAP accounts for 1 % of all colorectal cancers [19]. Patients with chronic inflammatory bowel syndrome (IBS), particularly ulcerative colitis and Crohn’s disease, have an increased risk of developing colorectal cancer [20]. Other risk factors include type 2 diabetes, being overweight or obese, physical inactivity, smoking and drinking alcohol. Consumption of a diet high in red meat or processed meat can increase the risk of colorectal cancer. Diets low in vegetables, fruits and whole grains have also been linked with an increased risk of developing colorectal cancer [21, 22].

Early detection is important in preventing colorectal cancer. People who have a strong family history of colorectal cancer polyps or cancer are advised to do genetic screening for HNPCC-associated gene mutation and FAP-associated gene mutation. Colonoscopy, flexible sigmoidoscopy, double-contrast barium enema, CT colonoscopy are performed every 5 years to detect the presence of colorectal polyps and cancer. Non-invasive tests such as fecal occult blood test (FOBT) can be done annually for the detection of colorectal cancer [23]. Depending on the stage and site of the tumour, treatment options can vary. Tumour resection surgery may be combined with radiation or chemotherapy as either adjuvant or neo-adjuvant therapy. 5-fluorouracil and Oxaliplatin are the most commonly used drugs among the many more combinations that can be used for treating colorectal cancer.

Despite advances in screening and surgical treatment, metastatic cancer has no known cure, and the 5-year survival rate is disappointingly low (approximately 8 %). Such alarming ineffectiveness of standard anti-cancer therapies has been attributed to the existence of relatively rare, highly drug-resistant, quiescent or slow proliferating cells with stem-like properties called CSCs [24, 25].

### Colorectal cancer stem cells

Although only a small subpopulation (<1 %) of overall cancer cells have the ability to proliferate extensively and form new tumours [26, 27], they are the crucial component responsible for tumour recurrence, therapy resistance and metastasis [28, 29]. CSCs may undergo symmetrical cell division into two identical daughter CSCs or asymmetrical cell division to give rise to one daughter CSC and one differentiated progenitor cell, resulting in a numbered expansion of CSCs as the tumour grows [30]. It has been recently suggested that CSCs may arise from normal stem cells, progenitor cells, or more differentiated cells [30, 31] through multiple mutations of genes as a result of genomic instability [32] or oncogene-induced plasticity [33].

CSCs are not synonymous with normal stem cells. They differ significantly from normal stem cells in their tumourigenicity; CSCs can form tumours when transplanted into animals, but normal stem cells cannot [30]. Thus, CSCs are defined by four key characteristics: (a) self-renewal—CSC populations can be serially transplanted through multiple generations, indicating they have self-renewing capacity; (b) differentiation—pluripotent cells can not only undergo symmetrical cell division to form tumourigenic daughter CSCs but can also generate bulk populations of non-tumourigenic cells by asymmetric cell division; (c) tumourigenicity—a small subpopulation of CSCs have tumourigenic potential when transplanted into animals and (d) specific surface markers, by which the CSC subpopulation can be separated from non-stem cells [28, 34, 35].

CSCs can be isolated in vitro by the expression of specific cell surface markers, signaling pathways, intracellular enzyme activities and sphere forming ability in a non-adherent medium [36, 7, 37, 38]. Common cell surface markers for colorectal CSCs include CD133, CD44, CD166, CD24, CD29, as well as Hoechst dye efflux or aldehyde dehydrogenase activity (ALDH), all of which characterize tumourigenicity and metastatic acquaintance, particularly through their ability to repopulate into colonies from a single cell [39, 40]. Additional assays are used to support the functional properties of CSCs characterized by their ability to show invasiveness, stemness properties and drug-resistance in in vitro assays. These include the 3-dimensional tumoursphere forming assay, invasion assay using Matrigel as well as the drug resistance assay for various chemo-/radio therapy agents [41–44]. However, in vitro assays alone are not enough to demonstrate the detection of CSCs, and in vivo assays are regarded as the gold standard, including serial transplantation in animal models [30]. This is the process in which tumour cells are transplanted into immunocompromised (typically NOD/SCID) mice, and tumour growth is monitored; then, xenograft tumours or primary tumours are isolated from the mice and implanted again into other immunocompromised mice to demonstrate self-renewal and tumour formation capacities [30, 45, 46].

### Curcumin: properties and its derivatives

Curcumin is a well-known dietary polyphenol derived from the rhizomes of turmeric (*Curcuma longa*), an Indian spice which is usually used in the preparation of mustard and curry [8, 47]. Curcumin is well-known for its anti-inflammatory, antioxidant, and antimicrobial activities [8, 10, 48], and it has been widely studied as a cancer chemopreventive agent in a wide range of cancer models, including head and neck, melanoma, brain, breast, colon, pancreatic, prostate and ovarian cancers, over the past three decades [48–50].

There are three curcuminoids, namely curcumin, demethoxycurcumin and bisdemethoxycurcumin, which are obtained from the natural extract of *Curcuma longa*, of which curcumin is the principal constituent [51]. Novel analogues of curcumin are also used as they dramatically improve the stability, bioavailability, and growth-inhibitory capacity, compared to curcumin, which has limited use in clinical applications due to its poor bioavailability [52, 53]. Furthermore, various nanotechnology-based formulations of curcumin have recently been considered for an innovative approach to overcoming the bioavailability and stability issues in brain tumour [54] and colorectal cancer [55].

## Effects of bioactive curcumin on cancer stem cells

During the past few years, a number of studies have suggested that curcumin has the potential to target CSCs through direct or indirect influences on the CSC self-renewal pathways [56–59]. Three major signaling pathways in which curcumin plays a pivotal role in CSC self-renewal behavior are the Wnt/β-catenin, Sonic Hedgehog (SHH), and Notch pathways [29]. A considerable number of in vitro and preclinical studies strongly support the potential use of curcumin as a cancer chemo-preventive agent. Therefore, numerous studies have been conducted to address the pharmacokinetics, safety and efficacy of curcumin in different types of cancer in human subjects. Published studies have generally reported no toxicity with a moderate dose of curcumin over a few months, and a number of studies have shown the beneficial effects of curcumin against a variety of pre-malignant or malignant disorders [60–66].

The mechanisms of CSC resistance to chemo- and radiation therapies and the potential targets for CSC-focused drug development have been extensively studied. It has been convincingly demonstrated that, in many cancers, the tumourigenic cells expressing common CSC markers, particularly CD133 and CD44, are not only resistant to commonly used anti-cancer drugs in colorectal cancer, including 5-FU and Oxaliplatin, but their number is also significantly increased after treatment [67–77]. Recently, much attention has been focused on several phytochemicals showing promising anti-cancer abilities/properties and specific CSC-targeted activities to help overcome the resistance and recurrence found with traditional therapies. Since CSCs are more resistant to conventional therapies in comparison with the differentiated cells constituting the tumour bulk, a combination of curcumin and conventional anti-cancer drug therapies may have the potential to overcome tumour resistance and reduce recurrence.

The efficacy of curcumin in targeting colorectal CSCs, which is summarised in Table 1, will be discussed in this section. Seven studies were selected for the review, two of which included an in vivo study in mice (S1 and S2). These two studies were conducted both in vitro and in vivo. In S1 by Lin et al., a new curcumin analogue, GO-Y030, was used to target colorectal CSCs [78]. Persistent activation of the STAT signal was seen in most of the cancers, including colorectal cancer, and GO-Y030 inhibited the phosphorylation of STAT3. Therefore, there was decrease in tumour formation and growth achieved via induction of apoptosis. The study was also conducted in vivo and there was a reduction in tumour growth, weight and mass by the same mechanism of STAT3 inhibition. Thus, it can be concluded that GO-Y030 is effective both in vitro and in vivo. In S2 by Wang et al, the encapsulated curcumin (CSO-SA micelles) was compared with free curcumin and empty CSO-SA [79]. Curcumin-loaded CSO-SA micelles were designed to achieve better stability and efficiency. They inhibited the CSC population by reducing the expression of CD44^+^/CD24^+^ markers and suppressed spheroid formation both in vitro and in vivo. Hence, the new formulation of curcumin can be more beneficial for bioavailability.

**Table 1:** Effect of curcumin and curcumin analogues in colorectal cancer stem cells: in vitro and in vivo (mice models) studies

Study	Authors	Title	Journal and year	In vitro study	In vivo study (Mice)	Experimental design	Results
1	Lin et al.	Targeting colon cancer stem cells using a new curcumin analogue, GO-Y030 [78]	British Journal of Cancer, 2011	Yes	Yes	ALDH+/CD133+ colon CSC were isolated from DLD1, HCT-116, and SW480 and HT29 colon CSC by flow cytometry. These cells were treated with GO-Y030 and cell death was observed by flow cytometry and tumourspheres were counted in the differentiating medium. These cells of SW480 and HCT-116 were injected subcutaneously into mice models and observed	GO-Y030 inhibited STAT 3 phosphorylation, cell viability, and tumoursphere growth of CSC in vitro. It suppressed tumour growth of CSCs from both SW480 and HCT-116 colorectal cancer cell lines in vivo in mice model
2	Wang et al.	Novel micelle formulation of curcumin for enhancing antitumour activity and inhibiting colorectal cancer stem cells [79]	International Journal of Nanomedicine, 2012	Yes	Yes	Cells obtained from cell cultures or xenograft tumours labeled with CD44-APC and CD24-FITC were treated with curcumin encapsulated in stearic acid-g-chitosan oligosaccharide (CSO-SA) and free curcumin and compared. Intravenously CSO-SA was injected into the mice	In vitro, CSO-SA showed anti-proliferative effects, 6× greater than free curcumin. In vivo, it suppressed tumour growth
3	Kanwar et al.	Difluorinated-curcumin (CDF): a novel curcumin analog is a potent inhibitor of colon stem-like cells [42]	National Institute of Health, 2011	Yes	No	Chemo-resistant cells of HCT-116 and HT-29 treated with 5FU + Ox alone or in combination with curcumin or CDF were compared. CDF showed more inhibition of transporter protein, growth factor receptor attenuation	CDF together with 5-FU + Ox was more potent than curcumin in reducing CD44, CD166 in chemo-resistant colon cancer cells by inhibition of growth, apoptosis induction and disintegration of colonospheres
4	Nautiyal et al.	Combination of Dasatinib and curcumin eliminates chemo-resistant colon cancer cells [43]	Journal of Molecular Signalling, 2011	Yes	No	Chemo-resistant cells of HCT-116 and HT-29 were treated with Dasatinib and curcumin. Dose comparison was done for Dasatinib with and without curcumin	The combination therapy of Dasatinib and curcumin showed better inhibition of cell growth, invasion, and colonosphere formation and reduced CSC population by reduced expression of CSC specific markers
5	Yinjie Yu et al.	Elimination of colon cancer stem-like cells by the combination of curcumin and FOLFOX [44]	Translational oncology, 2009	Yes	No	FOLFOX-surviving colon cancer cells of HCT-116 line were used with media containing FOLFOX or curcumin or combination to analyze the protein levels of CD44 and CD166	Treatment of FOLFOX surviving colon cancer cells with combination of curcumin and FOLFOX resulted in marked reduction of CSCs, reduction in colonospheres, increased expression of EGFR
6	Buhrmann et al.	Curcumin suppresses crosstalk between colon cancer stem cells and stromal fibroblasts in the tumour microenvironment: potential role of EMT [80]	PloS One, 2014	Yes	No	HCT-116 was co-cultured with MRC-5 cells in a high density microenvironment to mimic the CSC/fibroblast interactions in vivo and treated with 5-FU and/or curcumin in concentration-dependent manner	Co-cultured HCT-116 and MRC-5 cells showed synergistic interaction, indicated by the expression of molecules/proteins involved in tumour progression. Curcumin interferes with the cross-talk by interfering with their regulations/expressions
7	Roy et al.	Difluorinated-curcumin (CDF) restores PTEN expression in colon cancer cells by down-regulating miR-21 [81]	PloS One, 2013	Yes	No	Fu-OX resistant cells generated in HCT-116, HT-29 and SW620 and the expression of miR-21 and PTEN protein measured after CDF treatment Xenograft tissue from SCID mouse transfected with miR-21 was also analysed for the expression of miR-21 and PTEN for comparison purposes	CDF restores PTEN expression by down-regulating miR-21 expression in Fu-Ox resistant cells from the colonosphere population, which showed overexpression of miR-21 and decreased levels of PTEN prior to CDF treatment

Difluorinated-curcumin (CDF) was used to treat chemo-resistant colorectal cancer cells (FOLFOX-resistant cells) in S3 by Kanwar et al. [42]. The conventional chemotherapy used for colorectal cancer is 5-fluorouracil and Oxaliplatin (5-FU and Ox), the combination is called FOLFOX. There were chemo-resistant cell populations, called CSCs, and they were treated with a combination of CDF and FOLFOX. The results showed significant inhibition of CSCs with a combination of CDF and FOLFOX compared to curcumin with FOLFOX. Similarly, the study of the effect of CDF on the expression of miR-21 and PTEN, in which the inverse relationship of expression was shown to be associated with tumourigenicity in cancers such as pancreatic cancer, was taken further by Roy et al. in S7 [82, 83]. Treatment with CDF was shown to down-regulate miR-21, up-regulate PTEN expression, and subsequently inactivate the Akt pathway, marked by a reduction of pAkt in the colonosphere. The effect of curcumin treated cells on the cancer colony was not shown. The above three studies show that novel formulations of curcumin are more effective than natural curcumin in inhibiting colorectal CSCs due to their superior stability, better accumulation and enhanced therapeutic efficacy in vivo.

In S4, by Nautiyal et al. curcumin was combined with Dasatinib for the treatment of chemo-resistant colorectal cancer cells [43]. This combination treatment reduced tumour growth, colonosphere formation and extracellular invasion of the colorectal cancer cells. CSC markers: ALDH1, CD133, CD44 and CD 166 expression were found to be reduced by up to 80–90 %. Moreover, curcumin was shown to reduce the toxicity of Dasatinib, as it lessened the dosage of the latter required to kill the cancer cells. This study indicated that combination therapy is highly effective in inhibiting carcinogenesis, and that the incorporation of curcumin has a greater benefit with reduced drug toxicities. In S5 by Yu et al. curcumin was combined with FOLFOX to treat FOLFOX-resistant colorectal cancer cells [44]. The combined treatment markedly reduced the expression levels of CD166, CD44 and EGFR in FOLFOX-surviving cells. It also caused disintegration of colonospheres, and the combination therapy was shown to be more effective than conventional chemotherapy alone.

An important aspect of carcinogenesis involves the dynamic interactions between the cancer cells and their microenvironment. More recently, S6, Buhrmann et al., showed the role of the tumour microenvironment in tumour progression via the interaction of colorectal cancer cells in the stromal fibroblast, and the effect of curcumin treatment on these interactions [80]. The monolayer and high-density co-culture method was utilised to mimic the in vivo micro-environment of colorectal cancer cells in the stromal fibroblast. It was demonstrated that the cross-talk between the co-cultured cells synergistically promoted tumour activity, reflected by the activation of tumour-promoting factors, as well as metastatic activity, compared to the control monolayer cultures. This activity was significantly increased with 5-FU treatments, demonstrating the enrichment of CSC populations. However, curcumin was shown to dramatically inhibit these activities and sensitize them to 5-FU treatments.

**Fig. 1:**
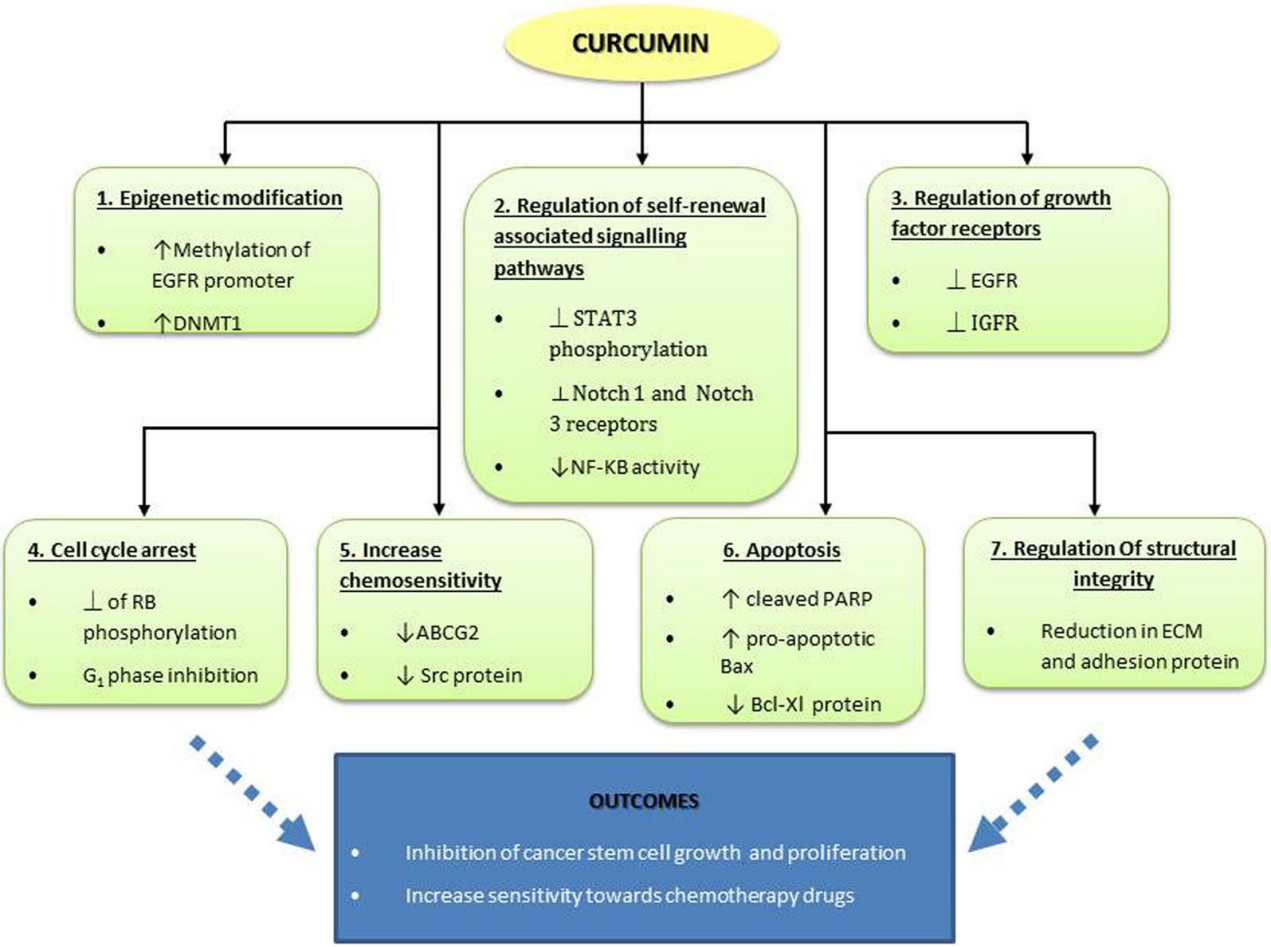
Effect of curcumin and curcumin analogue on colorectal cancer stem cells. Curcumin acts as an anti-tumour compound which targets the various factors or pathways that are implicated in colorectal cancer at many levels. Single or combined treatment of colorectal CSC has been shown to effectively inhibit tumour growth and, consequently, an improved sensitivity towards chemotherapy can be achieved. Key: *down arrow* down-regulate/decreased expression, *up arrow* up-regulate/increased expression, *perpendicular symbol* inhibit

In the light of the seven studies discussed, curcumin and its analogues target colorectal CSCs by multiple modulations at the molecular and cellular level, summarised in Fig. 1. Briefly, chemo-resistant colorectal CSC, either in vitro or in vivo, is effectively suppressed with curcumin treatment, either alone or in combination, by the complex mechanism of targeting intracellular targets such as epigenetic modification, miRNA regulation, as well cellular processes such as cell death, CSC-stromal cell interactions, and EMT in the CSC, thus effectively affecting the growth of the tumour cells. Taken together, curcumin effectively increases the sensitivity of the CSC either alone or when acting synergistically with chemotherapy drugs, thus overcoming CSC-associated drug resistance.

In a nutshell, curcumin, a naturally-occurring phytochemical, and its analogues were found to be effective in targeting chemo-resistant colorectal cancer cells. Modified formulations of curcumin were also synthesized to achieve better stability. Curcumin has been investigated in relation to many cancers and has proven to be a safe adjuvant or neo-adjuvant anti-cancer treatment. Here, it was studied in relation to the targeting of a small population of resident cells that are responsible for cancer recurrence in spite of the many advances in cancer treatment. These cells, known as CSCs play a major role in developing treatment resistance and tumour recurrence. Curcumin and its analogues significantly suppress CSCs both in vitro and in vivo, which can be seen by the reduced expression of CSC markers for colorectal cancer such as ALDH1, CD24, CD133, CD44, and CD166. Moreover, curcumin can be combined with conventional anti-cancer chemotherapies, such as 5-fluorouracil, Oxaliplatin and Dasatinib, to make the treatment more effective. With curcumin, the dose of chemotherapy can be lowered and, thus, drug toxicity is also reduced.

## Mechanism of action of curcumin on cancer cells and cancer stem cells

### Cancer stem cell related signaling pathways

In stem cells, normal proliferation, differentiation and cell renewal are controlled by a number of signaling pathways. Several studies have identified the key signaling pathways that play crucial roles in the growth and survival of stem cells from both normal and cancer tissue, such as Wnt/β-catenin, Notch, SHH and BMP signaling [36, 7]. Accumulating evidence has also shown the contribution of the PI3K/Akt pathway, implicated in the aggressiveness of CSC phenotypes [74, 84, 85]. In normal stem cells, self-renewal pathways play major roles in promoting proliferation and defining cell fate [29]. A large body of evidence has shown that the aberrant activation of these key regulatory pathways in cancer tissue, on the other hand, contributes towards the formation of CSCs and, therefore, leads to chemo-resistance, which causes the recurrence of tumour after chemotherapy treatment. Importantly, several studies have suggested cancer cells acquire stemness and drug resistance properties by the activation of the Wnt/β-catenin, Notch and SHH pathways [86]. Whether epithelial-mesenchymal transition (EMT), a key event implicated in the formation of CSC, is regulated via activation of the CSC related signaling pathways or induced by the tumour fibroblasts micro-niche remains to be elucidated.

**Fig. 2:**
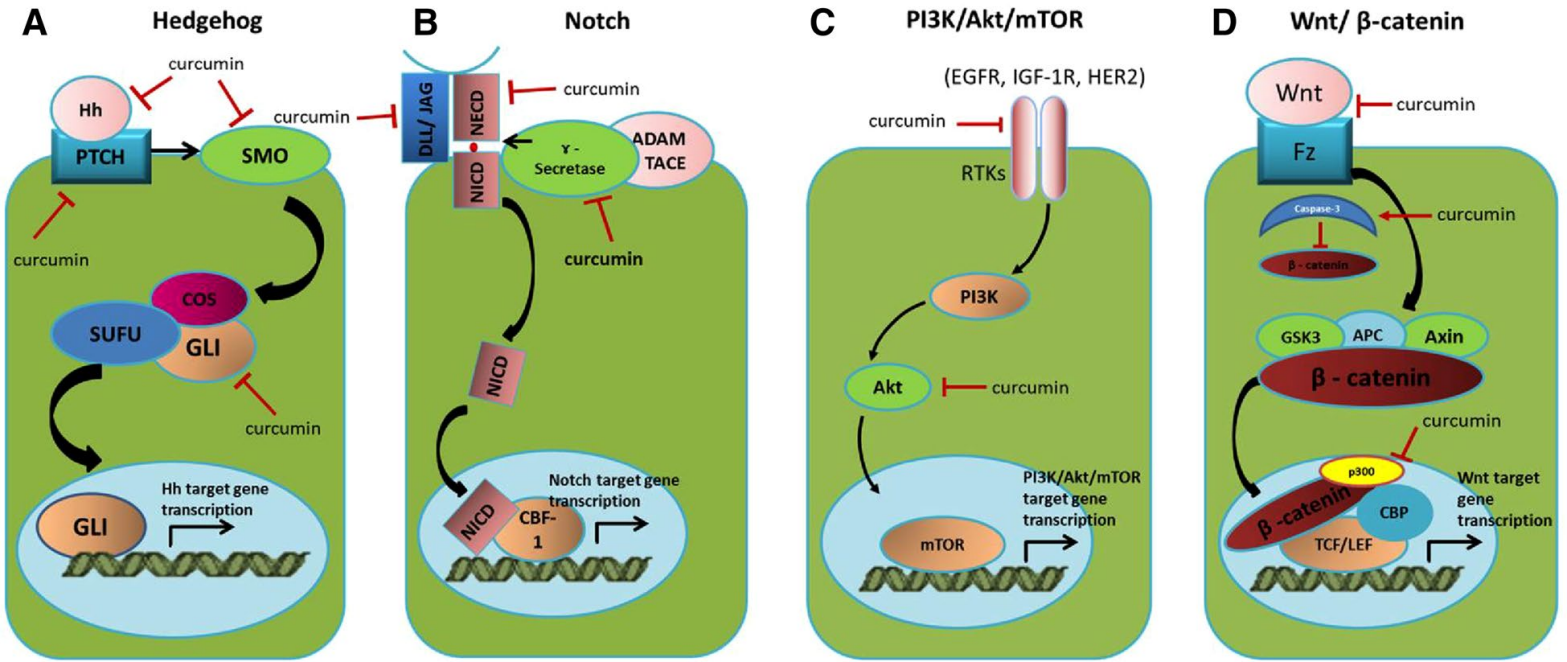
Mechanism of action of curcumin on cancer cells and cancer stem cells by targeting the self-renewal signalling pathways. Curcumin modulates the fate of cancer stem cell by targeting misregulated signalling pathways at multiple cellular levels namely; receptors, downstream effectors and transcriptional activity in **a** Hedgehog, **b** Notch, **c** PI3K/Akt/mTOR and **d** Wnt/β-catenin signalling pathways

Even so, theoretically, the CSC related pathways might be potential targets for cancer therapy, but in practice it is not an easy task due to the complex nature of signaling transduction and the involvement of curcumin effectively inhibiting activation of these pathways at the receptor level via multiple modes of action: inhibition of the ligand binding site of the receptor, inhibition of the formation of the receptor complex, and/or reduction in the abundance of the receptor [87–89], which is evidenced in all of these signaling pathways. Curcumin also effectively reduces the expression levels of the downstream effectors of these pathways at the mRNA and protein levels. This is evidenced by the reduction in the stability of β-catenin, the downstream mediator of Wnt/β-catenin signaling [90, 91], the reduction in Gli1 mRNA levels, which affects SHH signaling, [92] as well as inhibition of the phosphorylation of the downstream kinases signal transducers in the PI3K/Akt/mTOR signaling pathways [87, 93, 94]. At the transcriptional level, curcumin has been reported to reduce the components of the transcriptional complex, such as p300, that reduces the expression of the target genes [95, 96] and inhibits Gli1 transcriptional activity in the Shh pathway [97, 98]. The effect of curcumin and its analogues on CSCs and the self-renewing signaling pathways studied using both in vitro and in vivo experiments in various cancer types indicates the potency of this compound in enhancing the efficacy of the current cancer treatment modality, chemotherapy. This is particularly important owing to the effectiveness of these compounds in targeting CSCs at multiple levels of the signal cascade, which confers the survival, stemness and invasion properties of these cells and ensures the delivery of a more effective anti-cancer action by curcumin and its analogues. The mechanism by which curcumin and its analogues might be utilized in anti-cancer therapy, via the modulation of the signaling pathway, is illustrated in Fig. 2 and described in Table 2.

**Table 2:** Curcumin targets multiple levels of the cancer stem cell related signaling pathways

Cancer stem cell related signaling pathways	Curcumin targets multiple levels of signal transduction pathways	References
Hedgehog signaling	*Receptor*: Curcumin treatment led to a decrease in Shh and PTCH1 at protein level, triggering apoptosis in medulloblastoma cells	[92, 97, 98]
	*Downstream effector*: Down-regulation of Gli1 expression in curcumin treated-brain tumour cells	
	*Transcriptional activity*: Reduction in Gli1 mRNA level, therefore it downregulates Gli1-responsive genes	
Notch signaling	*Receptor*: (1) Curcumin downregulated Notch-1 at transcriptional level, which subsequently lowered the abundance of the receptor	[88, 89, 99]
	(2) Curcumin suppressed Notch-1 activation through down-regulation of a key component of the γ-secretase protein complex in esophageal cancer cells	
	*Transcriptional activity*: Curcumin inactivated nuclear factor-kB DNA-binding activity in oral carcinoma CAL-27 cells, thus targeting the cancer cells by activating apoptosis and reducing cell growth and invasion	
PI3K/Akt signaling	*Intermediate transducer*: Curcumin inhibited of the activation of PI3K/AKT/mTOR and its related pathways at multiple regulatory effectors via modulation of their phosphorylation status[Akt (T308 and S473), FoxO1 (S256), GSK3β (S9), tuberin/TSC2 (T1462), mTOR (S2448/2481), p70 S6K (T389), S6 (S235/236), 4E-BP1 (S37/46), eIF4G (S1108)]	[85, 87, 93, 94]
	*Transcriptional activity*: Curcumin decreased total expression of mTOR, Raptor and Rictor protein and mRNA levels	
Wnt/β-catenin signaling	*Receptor*: Frizzled-1 (the receptor of Wnt proteins) was inhibited in curcumin- treated human head and neck squamous cell carcinoma cell line mda 1986	[90, 91, 92, 95, 96]
	*Downstream effector*: Curcumin induced caspase-3-mediated cleavage of β -catenin, leading to inactivation of Wnt/β-catenin signaling	
	*Transcriptional activity*: Curcumin reduced β-catenin/tcf transcriptional activity via downregulation of β-catenin and its positive regulator, p300	

### Regulation of microRNAs

MicroRNAs (miRNAs) are a class of short highly conserved non-coding RNAs which have emerged as key post-transcriptional regulators of gene expression [100]. Numerous data have revealed that miRNA regulates a variety of biological events, including development, cell proliferation, differentiation, senescence and apoptosis [100–102].

**Fig. 3:**
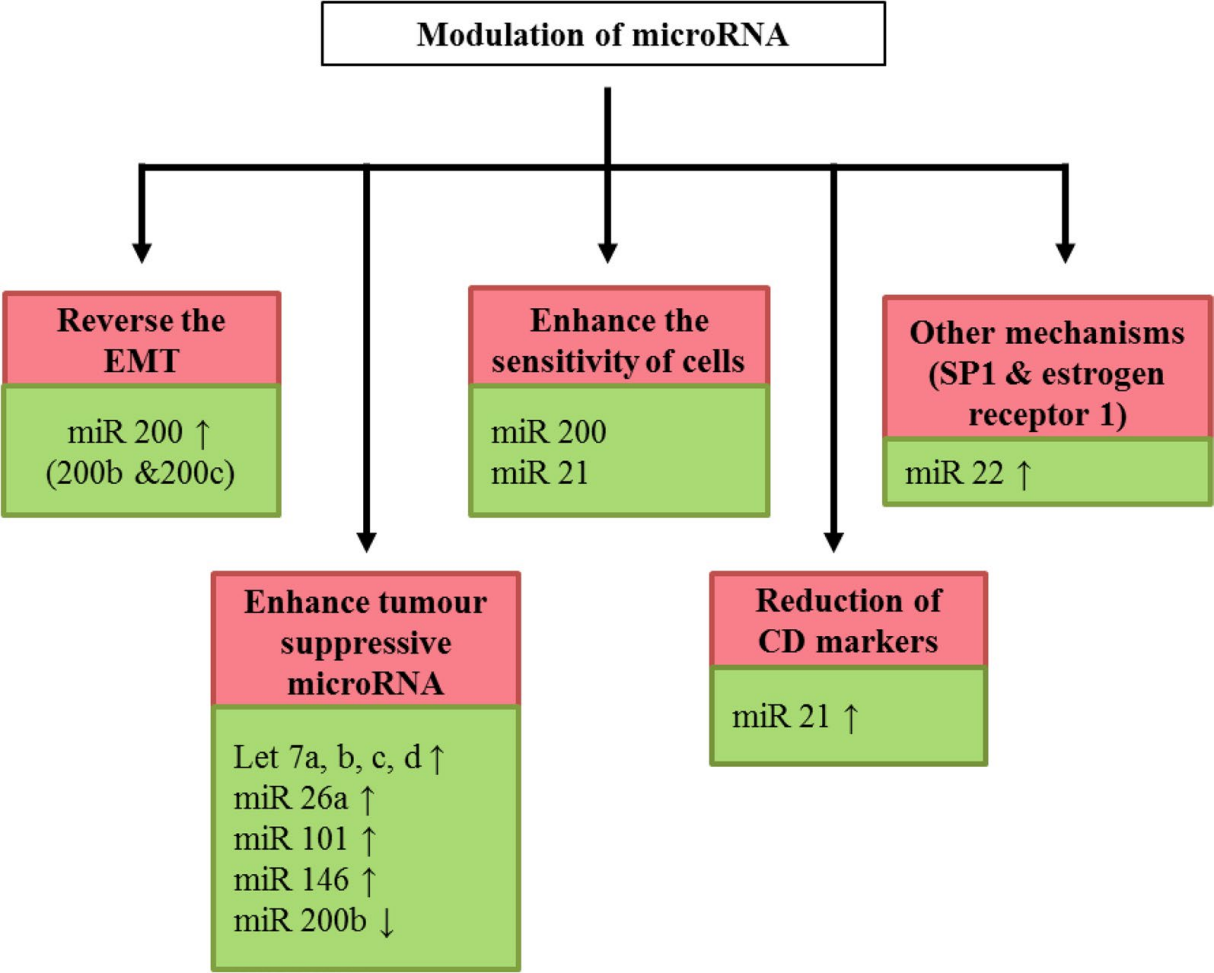
Mechanism of action of curcumin on cancer cells and cancer stem cells via modulation of expression of miRNA. Curcumin modulated expression of miRNAs are clustered into different mechanism of actions which involve the reversion of CSC phenotypes and properties and therefore, the cells are sensitised to the anti-cancer activity of curcumin

Only a few reports have so far investigated the effect of curcumin on miRNA expression. It was first found that miR-22 was the most up-regulated while miR-199a* the most suppressed in a human pancreatic carcinoma cell line treated with curcumin. The two downstream targets of miR-22, SP1 transcription factor (SP1) and oestrogen receptor 1 (ESR1), are implicated in promoting tumour development [103]. While the overexpression of SP1 protein contributes to metastasis in diverse tumour types, its inhibition in colorectal CSC has been shown to markedly suppress CSC growth and induce apoptosis, which can be achieved by treating the cells with curcumin. [104]. In another study, with a longer treatment period of 2 days, curcumin was found to promote human adenocarcinoma cell apoptosis through modulation of miR-186* and targeting its down-stream caspase-10 pathway [102]. Some miRNAs enhance the sensitivity of cancer cells to anti-cancer drugs and modulate their functionality via CD expression, such as miR-21 [105]. In human colorectal cancer RKO and HCT116 cells, curcumin inhibits the transcriptional regulation of miR-21 via AP-1, suppresses cancer cell proliferation, invasion and metastasis, and stabilises the expression of the tumour suppressor programmed cell death protein 4 (Pdcd4) [41]. Likewise, miR-200, which is up-regulated in cells treated with curcumin, enhances the sensitivity of cancer cells to anti-cancer drugs in addition to playing a key role in reversing EMT [106]. Furthermore, miRNAs have been reported to modulate tumour-suppressive mRNA. Some miRNAs have been identified that mask cancer cells from apoptosis, which is extremely important. Up-regulation of tumour-suppressive miRNAs, such as Let-7, miR-26a, miR-101 and miR-146, has been detected in cancer cells treated with curcumin [105, 106]. The role of curcumin as an anti-cancer therapy via the regulation of miRNA expression is depicted in Fig. 3.

### Epithelial-mesenchymal transition

Epithelial-mesenchymal transition (EMT) is a complex, highly regulated process during embryonic development in which certain epithelial cells undergo morphogenetic changes to become cells with mesenchymal cell-like properties. This process allows recruitment of the mesenchymal cells to migrate to a specific site in the developing embryo and consequently differentiate to form epithelial cells at a distal location via the reverse process, mesenchymal-epithelial transition (MET) [107]. The metastasis process during tumourigenesis progression has been associated with the initiation of the EMT process, where cancer cells acquire aggressive and invasive properties. The signaling pathways involved in developmental EMT have also been implicated in the self-renewal and maintenance of CSCs, suggesting that EMT has a role in the malignant progress of tumour cells caused by CSCs [108, 109].

The TGF-β pathway plays a crucial role in the regulation of transcriptional repressor genes during the initiation of EMT, such as Zeb1, Snail, Slug and Twist [108]. These transcription factors repress the expression of E-cadherin, activating cascades of the cellular events and genes driving the EMT [110]. The miRNA family is also an important regulator of the EMT process, particularly the miR-200 and the let-7 family, thus it may also contribute to the metastatic and drug resistance properties found in CSCs [111].

Decreased expression of the miR-200 family has been recognised as one of the signatures of invasiveness and metastasis progression in pancreatic cancer cells, hence indicating its regulatory role in tumour suppression [112]. Similarly, drug-resistant cancer cells which exhibited more EMT like properties were found to be low in the expression of miR-200 [113, 114]. Several studies have highlighted the possible mechanisms by which miR-200 suppresses formation of CSCs, through inhibition of the self-renewal pathway evidenced by the downregulation of Notch-1 and BMI1, reversion of EMT and regulation of a few other regulators.

The use of natural phytochemicals to manipulate the expression of miRNA has been proven effective in reversing the EMT process in CSCs by targeting the repressor proteins involved and promoting cancer cell apoptosis [115]. For example, experimental evidence has shown the feasibility of reversing the EMT phenotype in pancreatic cancer by means of blocking the Zeb1 protein and vimentin, which are highly expressed in mesenchymal cells, via RNAi modulation produced by in vitro treatment with chemical inhibitors such as isoflavones and curcumin [82, 83, 106, 115]. In another recent study, curcumin was shown to effectively sensitize colorectal CSCs towards chemotherapy drugs by blocking the synergistic effect of HCT116 cells in the fibroblast MRC-5 cell co-culture, which showed an increase in EMT-phenotypic expression compared to the HCT-116 cell monoculture [80]. Taken together, these studies suggest the possibility of inhibiting CSCs by targeting EMT, and curcumin has been proven to be one of the effective modalities.

The results of the aforementioned studies suggest that individual miRNA might play multiple roles in several human cancers during carcinogenesis and tumour progression. Additionally, the close relationship between miRNA regulation and the EMT signalling process could be further investigated with other repressor proteins that have active roles in the acquisition of the EMT phenotype in malignant CSCs. It can be assumed that curcumin has potential therapeutic properties in human cancer, mainly by targeting miRNA expression, and that it functions to induce cell apoptosis as well as to modulate drug resistance in cancer cells. These therapeutic properties of curcumin have been investigated and supported by the promising evidence from in vitro and preclinical studies, but the effect of curcumin in cancer therapy via miRNA modulation needs to be confirmed by more studies especially in vivo and clinical trials.

## Future perspective

**Fig. 4:**
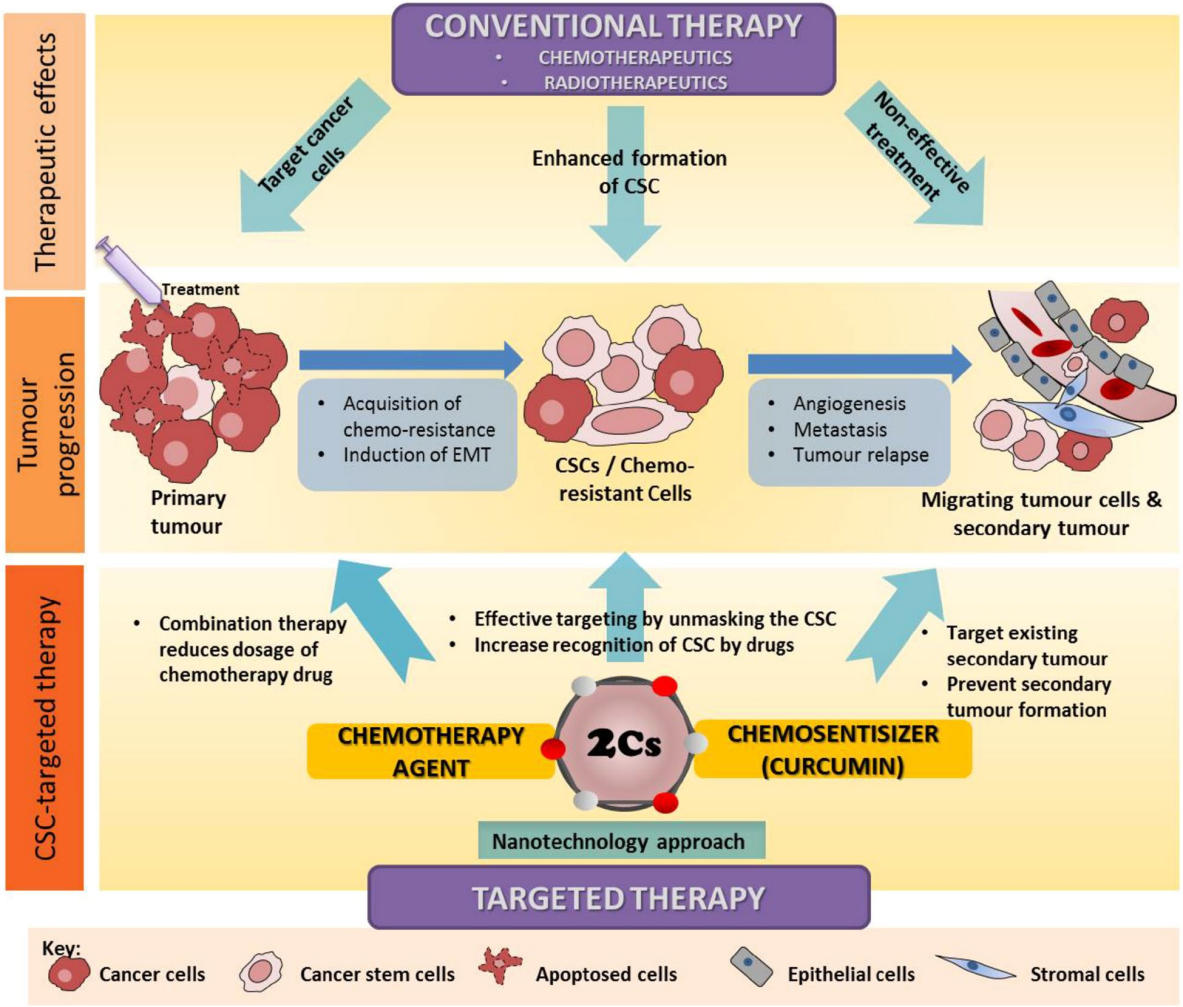
Schematic diagram of the outlook of advanced therapy for targeting cancer and resistant CSC incorporating nanotechnology approaches to improve the formulation of curcumin and its analogues to achieve better therapeutic effects. The *top panel* shows the typical effects of conventional therapy in relation to tumour progression (*middle panel*), noting that CSC typically escapes the treatment, thus causing tumour recurrence and metastasis. The *bottom panel* shows the future effect of targeting the CSC population using curcumin and its analogues and the future perspective of using a nanotechnology approach to improve drug formulation

Curcumin and its analogues reduced colorectal cancer stem cell populations through various mechanisms: affecting signaling molecules, apoptotic genes, growth factor receptors, tumour spheroid formation, microRNAs, and epithelial-mesenchymal transition. The effects were seen not only in in vitro but also in in vivo mice studies as well. Curcumin reduced tumour formation and recurrence in chemo-resistant cancer cells by targeting the cancer stem cell (CSC) population. The current limitations for the clinical application of curcumin and its analogues are due to their low absorption and stability, thus resulting in low cellular bioavailability. Novel formulations of curcumin and its analogues have been developed for better bioavailability and have been found to be effective in CSC inhibition. Conventional therapy, either chemo- or radiotherapy, specifically targets the rapidly dividing cells within the tumour mass. These therapies, however, fail to effectively eradicate the whole tumour mass, which may escape the treatment due to acquired chemo-resistance. This chemo-resistance is favoured by the tumour-stoma microniche, which plays a key role in EMT induction and subsequently makes the cells acquire CSC properties and metastasise via the blood stream to initiate a secondary tumour as the cancer progresses. Therefore, conventional therapies, even at higher doses, are non-effective in targeting CSC and metastasised cancer cells. Curcumin and its analogues, with their capacity to target both cancer cells and CSCs as well as increase the susceptibility of CSC towards chemo- and radiotherapy, therefore, have the potential to be developed as an adjuvant anti-cancer agent. Further investigation is underway to develop curcumin based anti-cancer therapy using nanotechnology approaches, such as the nanoparticle delivery system, to overcome the pharmacokinetic and pharmacodynamic drawbacks in order to develop an effective and targeted therapy for eradicating cancer (Fig. 4).

## Conclusion

Overcoming tumour recurrence remains a major challenge in the treatment of colorectal cancer, due to the presence of the CSC population which contributes to chemo-resistance. Curcumin and its analogues have been shown to effectively kill cancer cells, and increasing evidence has shown their potential in targeting CSCs via regulation of the signaling pathways, specific microRNAs and epithelial mesenchymal transition. Taken together, these compounds, with their promising anti-cancer and anti-CSC potential, can be further developed using better formulations to enhance the efficacy of existing treatment modalities.

## Abbreviations

Akt: apoptotic phosphoinositide 3-kinase
ALDH: aldehyde dehydrogenase
ABCG2: ATP-binding cassette of family G2
Bax: Bcl-2 associated X-protein
BMP: bone morphogenetic protein
CD: clusters of differentiation
CSC: cancer stem cell
EMT: epithelial-mesenchymal transition
CDF: difluoronated curcumin
CSO-SA: curcumin encapsulated in stearic acid-g-chitosan oligosaccharide
DNMT1: DNA 5-cytosine methyltransferase
ECM: extracellular matrix
EFGR: epithelial growth factor receptor
FAP: familiar adenomatous polyps
FOBT: fecal occult blood test
5-FU: 5-fluoruracil
FOLFOX: combination treatment of 5-fluorouracil and oxaliplatin
HNPCC: hereditary non-polyposis colon cancer
IBS: inflammatory bowel syndrome
IGFR: insulin growth factor receptor
NON/SCID: non-obese diabetic severe combined immunodeficiency
PARP: poly [ADP-ribose] polymerase 1
PTEN: phosphatase and tensin homologue
RB: retinoblastoma protein
SHH: Sonic Hedgehog
